# Diagnostic performance of PIVKA-II in identifying recurrent hepatocellular carcinoma following curative resection: a retrospective cohort study

**DOI:** 10.1038/s41598-024-59174-5

**Published:** 2024-04-10

**Authors:** Wenfeng Zhu, Weilong Wang, Wenjie Zheng, Xiaolong Chen, Xiaowen Wang, Juping Xie, Shijie Jiang, Haoqi Chen, Shuguang Zhu, Ping Xue, Xiaofeng Jiang, Hua Li, Genshu Wang

**Affiliations:** 1 Department of Hepatic Surgery, Liver Transplantation, The Third Affiliated Hospital of Sun Yat-Sen University, Guangzhou, 510630 China; 2 Guangdong Key Laboratory of Liver Disease Research, The Third Affiliated Hospital of Sun Yat-Sen University, Guangzhou, 510630 China; 3 Department of General Surgery, The First Affiliated Hospital of Guangdong Pharmaceutical University, Guangzhou, 510630 China; 4 Department of General Surgery, The Seventh Affiliated Hospital of Sun Yat-Sen University, Shenzhen, 518000 China; 5 Department of Hepatobiliary and Pancreatic Surgery, The Second Affiliated Hospital of Guangzhou Medical University, Guangzhou, 510630 China; 6 State Key Laboratory of Traditional Chinese Medicine Syndrome, Guangzhou, 510630 China; 7 Department of Liver Transplantation, Guangdong Provincial Hospital of Traditional Chinese Medicine, Guangzhou, 510630 China; 8 The Second Affiliated Hospital of Guangzhou University of Chinese Medicine, Guangzhou, 510630 China; 9 Chinese Medicine Guangdong Laboratory (Hengqin Laboratory), Hengqin, 519031 China

**Keywords:** Biomarkers, Diagnostic markers, Predictive markers

## Abstract

Protein induced by vitamin K absence or antagonist II (PIVKA-II) plays a critical role in the diagnosis of hepatocellular carcinoma (HCC), however, studies on its efficacy in diagnosing recurrent HCC were rarely found. A multicenter, retrospective, and observational study was conducted. During the overall follow-up of 5 years, HCC patients who had curative resection were monitored every 3 months in the first year post-surgery and every 6 months thereafter if no recurrence occurred. Tumor markers were collected at the diagnosis of recurrence for those with recurrence and at the last follow-up for those without recurrence. The median serum levels of PIVKA-II and AFP in the recurrence group were significantly higher than those in the non-recurrence group (PIVKA-II: 84.62 vs. 18.76 mAU/ml, *p* < 0.001; AFP: 4.90 vs. 3.00 ng/ml, *p* < 0.001) and there is a significant correlation between PIVKA-II and AFP (R = 0.901, *p* < 0.001). PIVKA-II showed better accuracy than AFP in the diagnosis of overall recurrent HCC (AUC: 0.883 vs. 0.672; *p* < 0.0001), but also in patients with negative PIVKA-II before curative resection (AUC: 0.878 vs. 0.680, *p* = 0.001). Clinician should pay more attention to serum PIVKA-II values when following patients after curative HCC resection to detect early recurrence.

*Clinical trial registration*: ChiCTR2300070874

## Introduction

Primary hepatocellular carcinoma (HCC) is one of the most common cancers in the world^1^ and the second leading cause of cancer-related deaths each year^2^. Despite the remarkable success of curative resection of HCC in recent years, the recurrence rate in 5-year ranging from 60 to 70% is a major challenge in the treatment of HCC patients^3–5^. As a traditional biological diagnostic marker for the recurrence of hepatocellular carcinoma, elevated Alpha fetal protein (AFP) after treatment indicated the recurrence of HCC^6^. However, the effectiveness of supervising AFP is contested and varies due to the different etiology of HCC^7^. In addition, several studies have shown AFP has a low specificity although with a high sensitivity^8^. This may result from dormant necrosis and inflammation also leading to an elevation in AFP level^9^. In clinical work, it has also been observed that a lot of HCC patients have specific recurrence after curative resection with low serum AFP level. Therefore, it is of great significance to explore new serum markers for early diagnosis and prevention of recurrence of HCC after curative resection.

Prothrombin induced by vitamin K absence-II (PIVKA-II), also known as des-γ-carboxy prothrombin, its incomplete carboxylation of one or more glutamate residues in its γ-carboxyl glutamate structure leads to the loss of normal coagulation function^10^. PIVKA-II cumulative in HCC patients due to the deficiency of vitamin K-dependent carboxylase in malignant hepatocytes results in insufficient carboxylation of the prothrombin precursor^11^. Several studies shown that PIVKA-II had a better efficiency than AFP in the early diagnosis of HCC^12,13^. However, the performance of PIVKA-II in detecting recurrent HCC after curative resection is rare.

Therefore, we carried out this retrospective study to determine the diagnostic accuracy of PIVKA-II compared with AFP in recurrent HCC after curative resection. In order to solve this issue, the serum levels of PIVKA-II and AFP were collected in patients with and without recurrent HCC after curative operation. The performance of PIVKA-II and AFP in diagnosis of recurrent HCC were assessed.

### Patients and methods

A multicenter, retrospective, and observational study from 3 centers was conducted: The Third Affiliated Hospital of Sun Yat-Sen University (Guangzhou, China, between January 2016 and June 2018), The Second Affiliated Hospital of Guangzhou Medical University (Guangzhou, China, between June 2016 and June 2018), The First Affiliated Hospital of Guangdong Pharmaceutical University (Guangzhou, China, between January 2017 and June 2018). Inclusion criteria: (1) Patients with primary HCC and had not received other anti-tumor therapy; (2) Patient underwent curative resection; (3) No restrictions on gender, age, and ethnicity. Exclusion criteria were: (1) HCC patients complicated with preoperative metastasis, portal vein carcinoma thrombus or bile duct carcinoma thrombus; (2) Patients with positive pathological margin; (3) Patients combined with primary tumors of other organs; (4) Patients take warfarin or vitamin K1; (5) Patients with acute hepatitis, embryoma of gonad or any other diseases that could cause elevated AFP. Patients with primary HCC underwent curative resection were re-examined every 3 months in the first year after surgery, and then every 6 months in the later period if the tumor had not recurred. Tumor markers (AFP and PIVKA-II) were collected at the time of diagnosis for recurrent patients, and those at the last follow-up were collected for patients without recurrence. The recurrent HCC was diagnosed according to the same criteria for primary HCC.

Criteria for curative resection of liver cancer.

Intraoperative criteria: ① No macroscopic cancer thrombus was observed in hepatic vein, portal vein, bile duct and inferior vena cava; ② No invasion of adjacent organs, no hilar lymph nodes or distant metastasis; ③ The liver incisal margin was more than 1 cm from the tumor boundary. If the incisal margin is less than 1 cm, but histological examination of the resection section of the liver shows no residual tumor cells, that is, the incisal margin is negative.

Postoperative criteria: ① Ultrasound, CT and MRI (two of which must be performed) were performed 2 months after surgery and no tumor lesions were found; ② If tumor marker(AFP or PIVKA-II) is elevated before surgery, the retest 2 months after surgery is required to be in the normal range.

Patients were divided into recurrence group and non-recurrence group according to whether the HCC has recurred. Baseline data like age, gender, clinical results and laboratory findings were collected. Serum AFP was detected by electrochemiluminescence with Roche Cobase601 fully automated immunoassay analyzer with a reference range of < 8 ng/ml. PIVKA-II was measured by chemiluminescence immunoassay (I4000, Abbott Laboratories, USA) and the reference range was < 40 mAU/ml. All reagents are original kits and operate in strict accordance with reagent instructions. All operations in the process of determination are carried out according to the laboratory indoor quality control documents. Quality control tests were carried out before, during and after the specimens were tested.

All data were analyzed by SPSS 25.0 software. The levels of PIVKA-II and AFP in different groups were analyzed. The results showed that PIVKA-II and AFP levels were skewed distribution. Two independent samples were used to compare the levels of AFP and PIVKA-II between different groups, and the sensitivity, specificity, correct index, coincidence rate, positive predictive value, negative predictive value and Kappa value of serum markers were calculated. At the same time, the appropriate reference range of AFP and PIVKA-II was determined by drawing receiver operating characteristic curve (ROC). The linear correlation analysis of AFP and PIVKA-II was carried out in all samples and primary liver cancer. *p* < 0.05 was considered as statistically significant difference.

This study followed the most recent ethical guidelines of the World Medical Association Declaration of Helsinki and was reviewed and approved by the Institutional Review Boards (IRB) of each participating center (No. II2023-021-01; No. 2023-IIT-8; No. 2023-ks-03). Written informed consent was waived because of the retrospective nature of the study and that there was no study-specific intervention beyond routine clinical care. Medical records of the included patients were anonymized and de-identified before analysis.

## Results

### Patient characteristics

**Figure 1:**
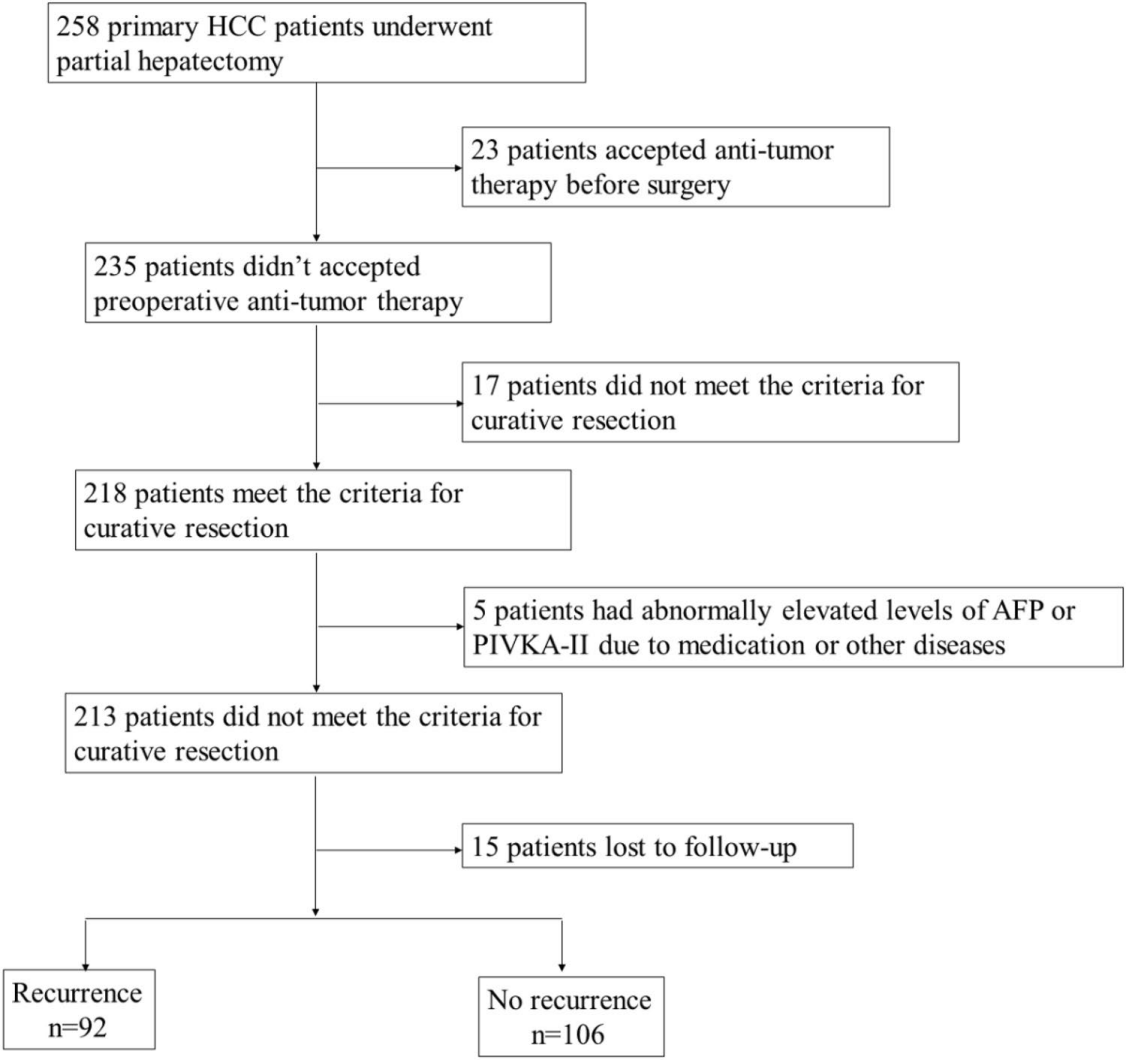
The flow chart of patient selection.

Of the 258 HCC patients that had a partial hepatectomy from the 3 centers included in the cohort, 23 were excluded from this cohort due to preoperative anti-tumor therapy. Of the remaining 235 patients, 17 were excluded from the study due to failing the criteria for curative resection, 5 patients were excluded due to abnormally elevated levels of AFP or PIVKA-II due to medication or other diseases, and 15 patients lost to follow up. Therefore, a total of 198 HCC patients were included in this study: 92 patients developed recurrent HCC after curative resection and 106 patients were found no sign of recurrence which considered as control group. The flow chart patient selection is shown in Fig. 1. There were 19 patients (20.65%) experienced local recurrence, while 73 patients (79.35%) experienced intrahepatic recurrence. A total of 70 patients (76.09%) had single tumors, and 22 patients (23.91%) had multiple tumors. Both recurrence group and non-recurrence group showed a male predominance, however, there was no significant difference in sex distribution between the two groups (*p* = 0.441). The later the BCLC stage, the higher the microvascular invasion (MVI) grade and the lower the degree of differentiation of tumor cell, and the more likely the recurrence in HCC patients after curative resection (*p* < 0.05). There is no significant correlation between postoperative recurrence of HCC and the cause of HCC (*p* > 0.05). The two groups were comparable in age, the levels of alkaline phosphatase (ALP), prothrombin time (PT), aspartate aminotransferase (AST), alanine aminotransferase (ALT) and the counts of white blood cell (WBC), red blood cell (RBC), platelet (PLT) (*p* < 0.05). Patients in recurrence group have a lower albumin (ALB) and higher glutamine transpeptidase (GGT) level than non-recurrence group (*p* > 0.05) (Table 1).

**Table 1:** Baseline characteristic of HCC recurrence and non-recurrence cases.

	Recurrence	Non-recurrence	*p*
Sex			0.441
Male	80 (40.4%)	88 (44.4%)	
Female	12 (6.1%)	18 (9.1%)	
Age*	55.15 ± 11.58	54.97 ± 10.51	0.909
MVI			< 0.001
MO	10 (10.9%)	51 (48.1)	
M1	43 (46.7%)	36 (34.0%)	
M2	39 (42.4%)	19 (17.9%)	
Differentiation			0.001
Low	47 (51.1%)	29 (27.4%)	
Moderate	30 (32.6%)	41 (38.7%)	
High	15 (16.3%)	36 (34.0%)	
Disease			0.161
Hepatitis B	38 (41.3%)	44 (41.5%)	
Hepatitis C	17 (18.5%)	32 (30.2%)	
Alcoholic cirrhosis	20 (21.7%)	14 (13.2%)	
Others	17 (18.5%)	16 (15.1%)	
BCLC			0.006
Stage 0	22 (23.9%)	40 (37.7%)	
Stage A1	27 (29.3)	39 (36.8%)	
Stage A2	43 (46.7%)	27 (25.5%)	
GGT (U/L)^#^	56.00 (32.00.97.75)	42.50 (27.00, 65.5)	0.001
ALP (U/L)^#^	82.50 (67.00, 106.75)	78.5 (62.75, 99.50)	0.277
ALB (g/L)^#^	38.2 (34.05, 42.20)	43.85 (39.45, 46)	< 0.001
PT (s)^#^	13.60 (13.10, 14.5)	13.6 (12.60, 14.70)	0.617
AST (U/L)^#^	35.00 (23.00, 55.50)	32.00 (22.75, 44.25)	0.227
ALT (U/L)^#^	34.00 (20.50, 58.25)	30.00 (21.00, 49.00)	0.539
WBC (10^9^/L)^#^	4.88 (3.85, 6.33)	5.26 (4.04, 6.47)	0.353
RBC (10^12^/L)^#^	4.75 (4.29, 6.21)	5.01 (4.59, 6.45)	0.104
PLT (10^9^/L)^#^	140.00 (99.00, 168.75.00)	152.00 (115.5, 185.00)	0.064

BCLC, Barcelona Clinic Liver Cancer; GGT, gamma glutamyl transferase; ALP, alkaline phosphatase; ALB, albumin; PT, prothrombin time; AST, aspartate transaminase; ALT, alanine transaminase; WBC, white blood cell; RBC, red blood cell; PLT, platelet.

*Values are expressed as means ± standard deviation.

^#^Values are expressed as median and interquartile ranges.

### PIVKA-II and AFP serum levels in recurrence and non-recurrence group

**Figure 2:**
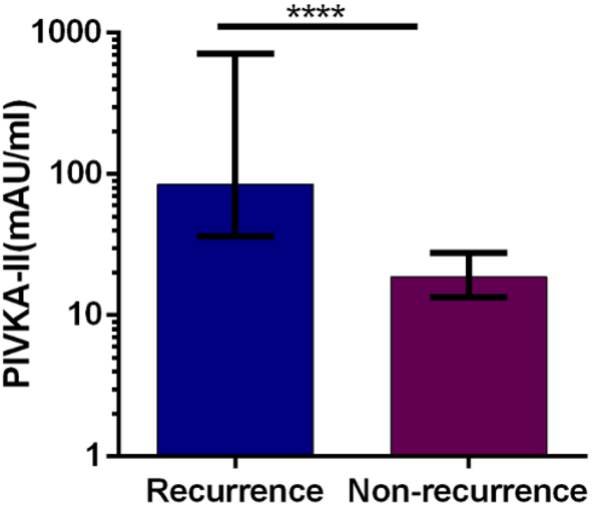
Median values of PIVKA-II in HCC recurrence patients and non-recurrence patients. Boxes and error bars refer to, respectively, the median value and the interquartile ranges in the two groups. Due to the data skewness, the levels of PIVKA-II and AFP are plotted on a log scale for better visualization. (“****” means *p* < 0.001).

**Figure 3:**
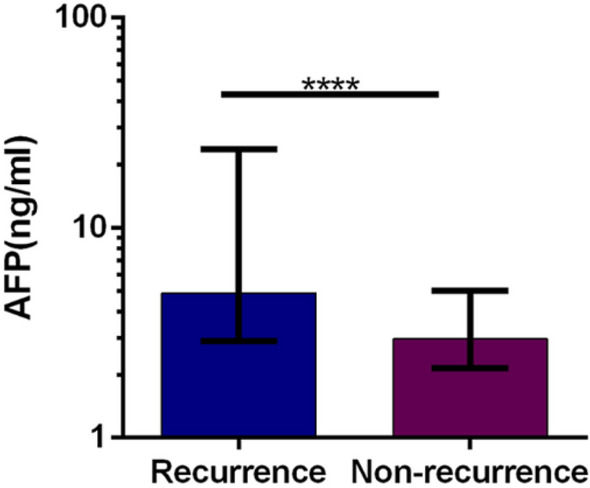
Median values of AFP in HCC recurrence patients and non-recurrence patients. Boxes and error bars refer to, respectively, the median value and the interquartile ranges in the two groups. Due to the data skewness, the levels of PIVKA-II and AFP are plotted on a log scale for better visualization. (“****” means *p* < 0.001).

The median serum levels of PIVKA-II and AFP in HCC recurrence group were significantly higher than those non-recurrence group. (84.62 vs. 18.76 mAU/ml, *p* < 0.001) (4.90 vs. 3.00 ng/ml, *p* < 0.001) (Figs. 2, 3).

### Relationship between PIVKA-II and AFP

**Figure 4:**
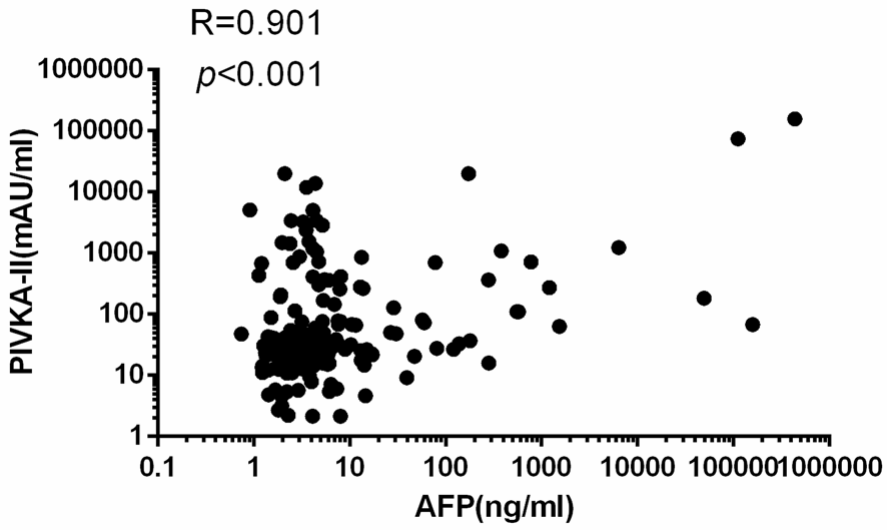
Correlation between PIVKA-II and AFP in all subjects. Correlation analysis was performed using Pearson correlation.

There is a significant correlation between PIVKA-II and AFP (R = 0.901, *p* < 0.001 for the Pearson correlation, Fig. 4).

### ROC curve of all patients

**Figure 5:**
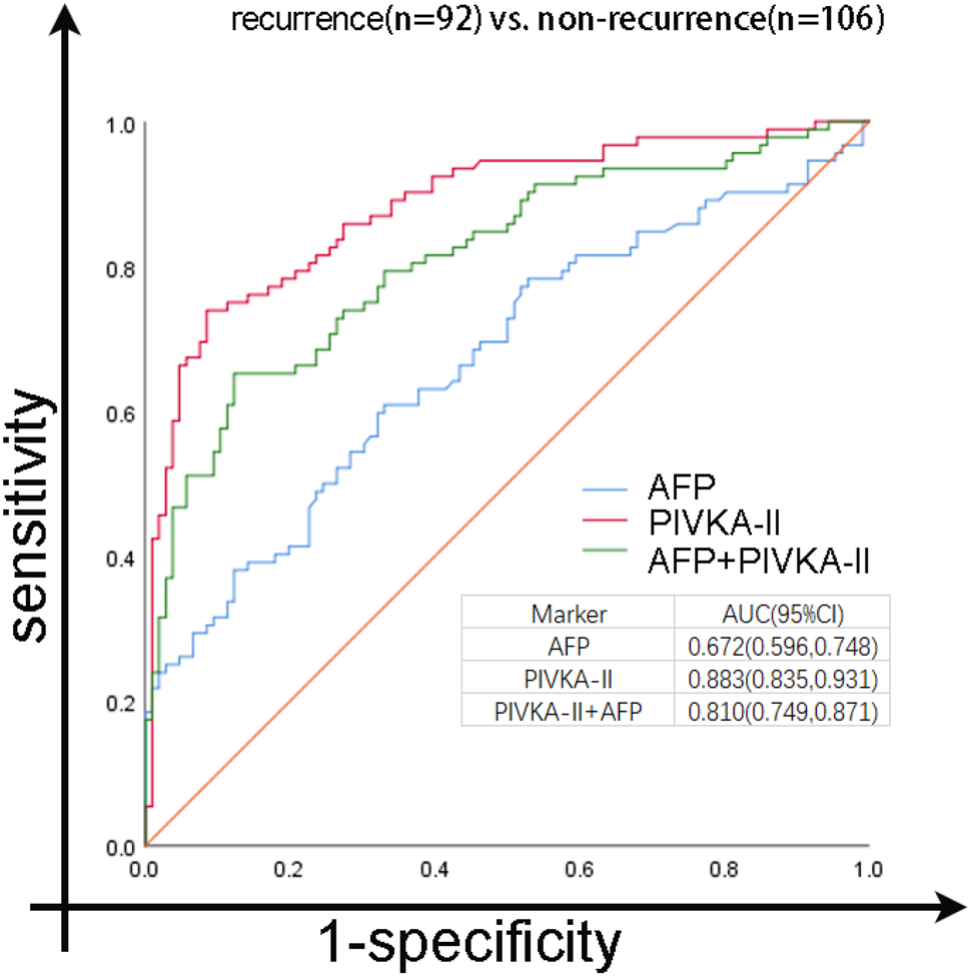
Receiver operating characteristics (ROC) curve comparing serum levels of PIVKA-II, AFP and a combination of PIVKA-II and AFP in HCC recurrence patients and non-recurrence patients. The area under the ROC curve (AUC) is shown with its 95% confidence intervals. PIVKA-II, prothrombin induced by vitamin K absence-II; AFP, a-fetoprotein.

In order to determine the cutoff values that would best distinguish recurrence patients from non-recurrence patients, ROC curves were plotted. A cutoff value of 40.84 mAU/ml for PIVKA-II and 7.40 ng/ml for AFP is optimal for identifying recurrence cases from non-recurrence cases. These values yielded sensitivity and specificity of 73.9% and 91.5% for PIVKA-II, and 38.0% and 87.7% for AFP, respectively. PIVKA-II showed a better accuracy than AFP in the early diagnosis of recurrent HCC (AUC 0.883; 95% CI 0.835–0.931 vs. 0.672; 95% CI 0.596–0.748, *p* < 0.0001) (Fig. 5). However, the combination of PIVKA-II and AFP did not improve the performance of diagnosis of recurrent HCC (Fig. 5).

### ROC curve in different groups

**Figure 6:**
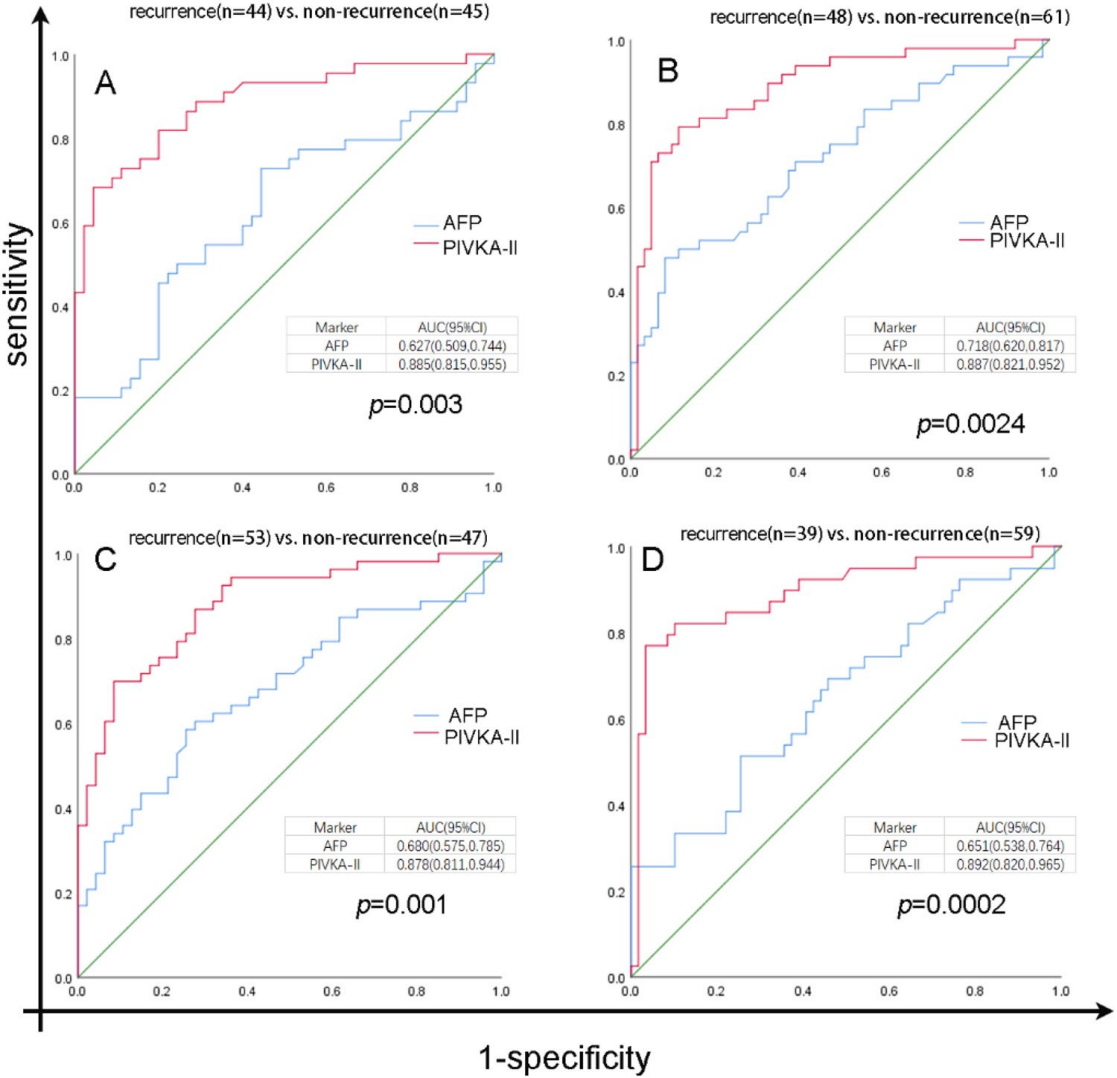
Receiver operating characteristics (ROC) curve comparing serum levels of PIVKA-II, AFP in HCC recurrence patients and non-recurrence patients in different groups. The area under the ROC curve (AUC) is shown with its 95% confidence intervals. PIVKA-II, prothrombin induced by vitamin K absence-II; AFP, alpha fetal protein.

In order to further explore the diagnostic efficacy of PIVKA-II in patients with HCC recurrence after curative resection, we further graded all patients. Patients were divided into several groups according to whether PIVKA-II or AFP was elevated when HCC was diagnosed before operation. (Group A: AFP < 8 ng/ml; Group B: AFP ≥ 8 ng/ml; Group C: PIVKA-II < 40.0 mAU/ml; Group D: PIVKA-II ≥ 40.0 mAU/ml). We redraw the ROC curve according to the new grouping. The diagnostic performance of PIVKA-II in group A was better than that of AFP (AUC (0.885; 95% CI 0.815–0.955 vs. 0.627; 95% CI 0.509–0.744, *p* = 0.003)) (Fig. 6A). In group B, PIVKA-II also showed better performance than AFP in diagnostic efficiency (AUC (0.887; 95% CI 0.821–0.952 vs. 0.718; 95% CI 0.620–0.817,* p* = 0.0024) (Fig. 6B). Similar results were also found in group C (AUC (0.878; 95% CI 0.811–0.944 vs. 0.680; 95% CI 0.575–0.785, *p* = 0.001)) (Fig. 6C) and group D (AUC (0.892; 95% CI 0.820–0.965 vs. 0.651; 95% CI 0.538–0.764, *p* = 0.0002)) (Fig. 6D). In other words, AFP did not show better performance in diagnosis of recurrent HCC than PIVKA-II when pre-operation AFP was elevated. PIVKA-II was demonstrated to have more accurate diagnostic efficacy than AFP when pre-operation PIVKA-II was elevated, even in patients with normal PIVKA-II range.

### Ethics approval and consent to participate

This study followed the most recent ethical guidelines of the World Medical Association Declaration of Helsinki and was reviewed and approved by the Institutional Review Boards (IRB) of each participating center (Ethics committee of the Third Affiliated Hospital of Sun Yat-sen University, No. II2023-021-01; Ethics committee of the First Affiliated Hospital of Guangdong Pharmaceutical University, No. 2023-IIT-8; Clinical Research Ethics Committee of the 2nd Affiliated Hospital of Guangzhou Medical University, No. 2023-ks-03). Written informed consent was waived because of the retrospective nature of the study and that there was no study-specific intervention beyond routine clinical care.

## Discussion

In this multicenter retrospective study, we mainly investigated the performances of AFP and PIVKA-II in the diagnosis of recurrent HCC after curative resection. Both AFP and PIVKA-II can be used to detect the recurrence of HCC, but PIVKA-II showed a better performance of discrimination between recurrence patients and non-recurrence patients. In addition, AFP did not show better performance in diagnosis of recurrent HCC than PIVKA-II when pre-operation AFP was elevated. PIVKA-II was demonstrated to have more accurate diagnostic efficacy than AFP when pre-operation PIVKA-II was elevated, even in patients with normal PIVKA-II range.

To date, the role of PIVKA-II in HCC has been widely studied, ranging from the diagnosis of early HCC^12,14,15^ to independent prognostic factors in HCC patients^16^, and to the prediction of MVI^12^. However, researches about the performance of PIVKA-II in diagnosing HCC of different causes and different stages shows diverse results. Ertle et al. proved that PIVKA-II mainly showed better performance compared with AFP in the diagnosis of HCC among non-cirrhotic patients^17^. Another study from Korea confirmed similar results in HCC with chronic hepatitis B^18^. Same finds was also verified by Li et al.^19^ In HCC patients with hepatitis C virus (HCV) and nonalcoholic fatty liver disease, PIVKA-II also showed superior performance than AFP^13,20^. In addition, although most of studies insist PIVKA-II was more sensitive than AFP for the diagnose of overall or early HCC^12,14,21,22^, adverse consequences also had been demonstrated that AFP was more efficient than PIVKA. Besides, serum PIVKA-II value ≥ 55 mAU/ml was demonstrated to identify patients with cirrhosis at higher risk of HCC development^20^. Also, PIVKA-II can be considered as independent predictors of overall survival (OS)^23^. However, there is still a lot of uncertainty surrounding how PIVKA-II is produced in HCC, and various factors like Vitamin K2 and g-glutamyl carboxylase may have complex relationships with PIVKA-II production^24^. Some clear evidence does indicate that serum PIVKA-II values are closely related to the occurrence and progression of HCC. PIVKA-II was proved to induces the expression of vascular endothelial growth factor (VEGF), which strongly related to potent mitogenic and migrative activities in angiogenesis of HCC^25–27^. Furthermore, PIVKA-II plays a critical part in the proliferation of HCC cells. Suzuki et al. reported that purified PIVKA-II significantly enhanced the DNA synthesis in PIVKA-II-producing cells rather than non-PIVKA-II-producing cell lines^28^. On the other hand, Ma et al. showed decreasing the secretion level of PIVKA-II by adding vitamin K2 in HCC cell lines significantly inhibit the proliferation^29^. They also confirmed the similar results in vivo by established xenograft tumor growth. The weight of the tumor decreased significantly as the serum level of PIVKA-II decreased by vitamin K2 in the same cell lines. These researches revealed that PIVKA-II act as growth factor in HCC and affect the proliferation of HCC cells. The underling mechanism of how PIVKA-II stimulate HCC angiogenesis remains unknown. PIVKA-II was found to contain two kringle domains which act as a member of angiogenic growth factor in HCC cells and required for HGF to bind to c-Met (the HGF receptor). PIVKA-II was proved to participate the signaling pathway (PIVKA-II-Met-JAK1-STAT3) for expression of angiogenic factors in HCC cells^25^. AFP has been regarded as an important indicator of HCC recurrence in the past and mounting evidence recently proved PIVKA-II as a useful diagnostic biomarker for HCC. Generous studies have focused on PIVKA-II in the first diagnosis of HCC and prediction of HCC recurrence, however, rarely research about PIVKA-II on the diagnosis of recurrence HCC after curative resection was found according to our literature review. Our study is the first to compare the performance of PIVKA-II and AFP in the diagnosis of recurrence HCC after curative resection. As our study showed, both AFP and PIVKA-II in the recurrence group were significantly higher than those in the non-recurrence group. Besides, correlation analysis showed that serum AFP value was positively correlated with PIVKA-II value. Interestingly, PIVKA-II not only showed a better accuracy than AFP in the diagnosis of overall recurrence HCC, but also in patients with negative PIVKA-II before curative resection. Surprisingly, the diagnostic accuracy in recurrence HCC of PIVKA-II was not increased when combined with AFP, which different from early diagnosis of HCC. The reason mainly ascribes to that most of the patients included in our study were patients with recurrent HCC, and the cause of HCC was mostly viral hepatitis B.

However, our study had several limitations. First, despite this is a multicenter study, the total number of patients enrolled in was small and the selection bias was still difficult to avoid. It is necessary to conduct a prospective study to compare the performance of PIVKA-II and AFP serum levels in detect the recurrence of HCC after curative resection. A second limitation is that this study was conducted in China, where most patients with HCC were suffering from HBV-related cirrhosis, that’s different from United States and Europe, where hepatitis C and excessive alcohol consumption are the leading etiologies of HCC.

In conclusion, our study confirmed that both AFP and PIVKA-II were able to detect the recurrence of HCC, however, PIVKA-II showed better performance than AFP. Although the combination of PIVKA-II and AFP did not improve the performance in detection of recurrent HCC. In addition, AFP did not show better performance in diagnosis of recurrent HCC than PIVKA-II when pre-operation AFP was elevated. PIVKA-II was demonstrated to have more accurate diagnostic efficacy than AFP when pre-operation PIVKA-II was elevated, even in patients with normal PIVKA-II range. Clinicians should pay more attention to serum PIVKA-II values when following patients after curative HCC resection to early detect recurrence, even in patients with normal preoperative PIVKA-II ranges.

## Data Availability

Data in this study are available from the corresponding author (wgshforsubmit@163.com).

## Abbreviations

PIVKA-II: Protein induced by vitamin K absence or antagonist II
AFP: Alpha fetal protein
HCC: Hepatocellular carcinoma
ROC: Receiver operating characteristic curve
IRB: Institutional Review Boards
BCLC: Barcelona clinic liver cancer
MVI: Microvascular invasion
ALP: Alkaline phosphatase
PT: Prothrombin time
AST: Aspartate aminotransferase
ALT: Alanine aminotransferase
WBC: White blood cell
RBC: Red blood cell
PLT: Platelet
ALB: Albumin
GGT: Glutamine transpeptidase
OS: Overall survival
